# Expression and secretion of a lytic polysaccharide monooxygenase by a fast-growing cyanobacterium

**DOI:** 10.1186/s13068-019-1416-9

**Published:** 2019-04-01

**Authors:** D. A. Russo, J. A. Z. Zedler, D. N. Wittmann, B. Möllers, R. K. Singh, T. S. Batth, B. van Oort, J. V. Olsen, M. J. Bjerrum, P. E. Jensen

**Affiliations:** 1 Copenhagen Plant Science Centre, Department of Plant and Environmental Sciences, University of Copenhagen, Frederiksberg C, Denmark; 2 Department of Geosciences and Natural Resource Management, University of Copenhagen, Frederiksberg C, Denmark; 3 Department of Chemistry, University of Copenhagen, Copenhagen, Denmark; 4 Novo Nordisk Foundation Center for Protein Research, Faculty of Health and Medical Sciences, University of Copenhagen, Copenhagen, Denmark; 5 Biophysics of Photosynthesis, Department of Physics and Astronomy, Faculty of Sciences, Vrije Universiteit Amsterdam, Amsterdam, Netherlands

**Keywords:** Cyanobacteria, Lytic polysaccharide monooxygenase, Bacterial secretion, *Synechococcus elongatus* UTEX 2973, Twin-arginine-translocation, General secretory pathway, *Tf*AA10A

## Abstract

**Background:**

Cyanobacteria have the potential to become next-generation cell factories due to their ability to use CO_2_, light and inorganic nutrients to produce a range of biomolecules of commercial interest. *Synechococcus elongatus* UTEX 2973, in particular, is a fast-growing, genetically tractable, cyanobacterium that has garnered attention as a potential biotechnological chassis. To establish this unique strain as a host for heterologous protein production, we aimed to demonstrate expression and secretion of the industrially relevant *Tf*AA10A, a lytic polysaccharide monooxygenase from the Gram-positive bacterium *Thermobifida fusca.*

**Results:**

Two variations of *Tf*AA10A were successfully expressed in *S. elongatus* UTEX 2973: One containing the native N-terminal, Sec-targeted, signal peptide and a second with a Tat-targeted signal peptide from the *Escherichia coli* trimethylamine-*N*-oxide reductase (TorA). Although the TorA signal peptide correctly targeted the protein to the plasma membrane, the majority of the TorA-*Tf*AA10A was found unprocessed in the plasma membrane with a small fraction of the mature protein ultimately translocated to the periplasm. The native Sec signal peptide allowed for efficient secretion of *Tf*AA10A into the medium with virtually no protein being found in the cytosol, plasma membrane or periplasm. *Tf*AA10A was demonstrated to be correctly cleaved and active on the model substrate phosphoric acid swollen cellulose. Additionally, expression and secretion only had a minor impact on cell growth. The secretion yield was estimated at 779 ± 40 µg L^−1^ based on densitometric analysis. To our knowledge, this is the highest secretion yield ever registered in cyanobacteria.

**Conclusions:**

We have shown for the first time high-titer expression and secretion of an industrially relevant and catalytically active enzyme in *S. elongatus* UTEX 2973. This proof-of-concept study will be valuable for the development of novel and sustainable applications in the fields of bioremediation and biocatalysis.

**Electronic supplementary material:**

The online version of this article (10.1186/s13068-019-1416-9) contains supplementary material, which is available to authorized users.

## Background

Cyanobacteria are versatile prokaryotic photosynthetic organisms that have attracted increasing interest as sustainable chassis for future biotechnological applications. Their potential has been demonstrated for the production of a wide range of biomolecules such as isoprenoids and next-generation biofuels (see recent reviews [1–7]). Fast growth is one of the essential features for biotechnological chassis. Therefore, major efforts have been put into finding and characterizing fast-growing cyanobacteria [8, 9]. One recently described freshwater cyanobacterial strain, *Synechococcus elongatus* UTEX 2973, is increasingly receiving attention due to its fast growth and genetic tractability. The strain is a close relative to the model cyanobacterium *Synechococcus elongatus* PCC 7942. They are 99.8% identical differing only in 55 single-nucleotide polymorphisms (SNPs), a 188.6 kilo base (kb) inversion and a 7.5 kb deletion/insertion [9]. SNPs in three specific genes, *atpA*, *ppnK*, and *rpaA*, enable three times higher biomass accumulation and tolerance to higher light intensities, temperature, and CO_2_ concentrations [10]. A recent study has reported doubling times of 1.5 h at 1500 µmol m^−2^ s^−1^ light, 5% CO_2_ and 42 °C, a rate comparable to some model heterotrophs [11]. *S. elongatus* UTEX 2973 has also been tested for its biotechnological potential. For example, the introduction of the sucrose transporter CscB led to a sucrose secretion rate of 35.5 mg L^−1^ h^−1^ [12] and genome-wide transcriptomes and fluxomes have been mapped [13, 14] which will facilitate future engineering efforts. Additionally, the development of genetic tools such as CRISPR-mediated gene editing make *S. elongatus* UTEX 2973 a promising option for photosynthetic biotechnology [15, 16].

To develop a novel production chassis, one important aspect is to be able to express heterologous proteins at high titers and to target them to a desired intra- or extracellular location. Targeting to the outer membrane has been successfully demonstrated with several reports of surface display systems established in cyanobacteria [17–20]. However, in some cases, it is useful to secrete the protein out of the cell into the culture medium. In diderm Gram-negative bacteria such as cyanobacteria, translocation of native proteins across the plasma membrane can occur via the twin arginine translocation (Tat) or the general secretion (Sec) pathways [21]. The Sec and Tat pathways, however, can only translocate proteins to the periplasm and requires engagement with further molecular machinery to effectively translocate proteins to the extracellular environment. Therefore, throughout this article, we will refer to translocation of a protein across the plasma membrane, to the periplasm, as “export” and the active transport of a protein from the interior to the exterior of the cell as “secretion” [22]. Although protein export and secretion pathways in cyanobacteria have been studied for almost two decades, they remain poorly understood (see recent reviews [21, 23]). Proof-of-concept studies have shown that heterologous protein secretion in cyanobacteria is possible, however, these studies have generally utilized native signal peptides fused to reporter proteins and have suffered from low yields [24, 25]. In this work, we engineered an industrially relevant extracellular enzyme, a lytic polysaccharide monooxygenase (LPMO), into the fast-growing cyanobacterium *S. elongatus* UTEX 2973 to assess its secretion capabilities. LPMOs are soluble, extracellular enzymes that act synergistically to boost the activity of other lignocellulosic biomass degrading enzymes [26–28]. Their active site contains a single copper ion which is coordinated by two histidine residues. Of these, the N-terminal histidine is both fully conserved and essential for activity [29]. LPMOs can utilize O_2_ or H_2_O_2_ to insert one hydroxyl group into the polysaccharide chain to break the glycosidic bond. The oxidation can occur at the C1 or the C4 position and the resulting products are oxidized oligosaccharides alongside their non-oxidized counterparts. For this work, we selected the secreted LPMO *Tf*AA10A from the Gram-positive *Thermobifida fusca* as our model for cyanobacterial secretion studies [30]. *Tf*AA10A was expressed with its native, Sec-targeted, signal peptide and, in a different construct, with a Tat-targeted signal peptide from the *Escherichia coli* trimethylamine-*N*-oxide reductase (TorA) replacing the native signal peptide. We show that a LPMO from a Gram-positive bacterial origin can be efficiently expressed and secreted into the culture medium by *S. elongatus* UTEX 2973. The secreted LPMO is active on a model cellulosic substrate and only a small difference in growth was observed in the generated strains. To our knowledge, this study presents the first evidence of heterologous enzyme secretion in the novel fast-growing cyanobacterium *S. elongatus* UTEX 2973 which constitutes a breakthrough in cyanobacterial secretion.

## Materials and methods

### Cultivation of cyanobacteria

All generated *S. elongatus* UTEX 2973 strains were maintained on BG-11 medium [31] supplemented with 10 mM 2-tris(hydroxymethyl)-methyl-2-amino 1-ethanesulfonic acid (TES) buffer (pH 8.0) and 1.5% (w/v) bacto agar at 30 °C with continuous illumination of approximately 75 µmol photons m^−2^ s^−1^. Liquid cultures were grown in P4-TES CPH medium, a modified version of phosphate-replete (P4) medium [32] where the component NaHCO_3_ was replaced for 10 mM TES buffer (pH 8.0). Liquid cultures were grown in glass tubes at 37 °C, bubbled with 3% CO_2_-supplemented air and continuous illumination of approximately 150 µmol photons m^−2^ s^−1^ (if not specified otherwise). For all strains containing a plasmid, liquid cultures were supplemented with 50 µg mL^−1^ spectinomycin and agar plates with 100 µg mL^−1^ spectinomycin. If not stated otherwise, cultures were induced with 1 mM isopropyl β-d-1-thiogalactopyranoside (IPTG) for 48 h before harvesting.

### Construct design and plasmid generation

The full-length amino acid sequence from *Tf*AA10A from *T. fusca* (Uniprot KB: Q47QG3) was codon-optimized for using GeneDesigner 2.0 [33]. A DNA sequence encoding a C-terminal human influenza hemagglutinin (HA) epitope tag [amino acid (AA) sequence: YPYDVPDYA] was added for protein detection purposes. The DNA sequence was custom-synthesized by GenScript (USA) and inserted into the RSF1010-based, self-replicating plasmid pDF-trc [34], containing a synthetic widely used trc promoter for gene expression, by standard restriction digest cloning (20 µL reaction), with the restriction enzymes *Eco*RI and *Hin*dIII (New England Biolabs), followed by ligation with T4 DNA ligase (Roche) according to manufacturer’s instructions. The generated plasmid was named pDAR3. A second plasmid was generated by overlap extension PCR cloning [35] where the DNA sequence encoding the native Sec signal peptide (AA 1–36) of *Tf*AA10A was replaced with a codon-optimized sequence of the N-terminus (AA 1–42) from trimethylamine-*N*-oxide reductase 1 (TorA) from *E. coli* (Uniprot KB: P33225). The generated plasmid was named pDAR21. All generated plasmid sequences were confirmed by Sanger sequencing. For all cloning steps, *E. coli* strain NEB^®^ 5-alpha (New England Biolabs) was used for plasmid amplification.

### Transformation of cyanobacteria and colony PCR

Plasmids were introduced into *S. elongatus* UTEX 2973 by biparental mating with an *E. coli* helper strain using standard procedures. In brief, chemically competent *E. coli* HB101 cells carrying a pRL443 helper plasmid [36] were transformed with the plasmid of interest. *E. coli* cells in exponential growth were harvested and mixed with 800 µL *S. elongatus* UTEX 2973 cells [optical density (OD) at 750 nm = 1]. The mixture was incubated overnight on a cellulose acetate/cellulose nitrate filter piece on a BG-11 agar plate [supplemented with 5% (v/v) lysogeny broth (LB)] at 30 °C in dim light. Cells were subsequently re-plated onto BG-11 agar plates containing 50 µg mL^−1^ spectinomycin and incubated in standard conditions (“Cultivation of cyanobacteria” section). After approximately 3 days, single colonies were re-streaked on agar plates containing 100 µg mL^−1^ spectinomycin and analyzed for the presence of the introduced plasmid by colony PCR. Colony PCR was performed using 2 µL of extracted total DNA from cyanobacterial colonies in a 25 µL standard PCR reaction with Q5 Hot-Start High-fidelity polymerase (New England Biolabs) and primers trc-F and trc-R following manufacturer’s recommendations (Additional file 1: Figure S1). DNA fragments were amplified using the following PCR conditions: 98 °C 3 min, 30 cycles of 98 °C for 2 min, 60 °C for 30 s and 72 °C for 1 min, 72 °C 1 min.

### Preparation of cleared cell lysates

For cell lysis, whole cultures were centrifuged at 4,000×*g* and resuspended in an appropriate volume of lysis buffer [20 mM Tris–HCl pH 7.5, 5% glycerol, 1% Triton X-100, complete protease inhibitor cocktail (Roche)]. Cells were broken with zirconium oxide beads (diameter: 0.15 mm) using a Bullet Blender Storm 24 (Next Advance) with the following settings: 3 times 5 min intervals at level 10, 12, 10. The lysates were centrifuged at 10,000×*g* for 10 min at 4 °C to remove cell debris and unbroken cells. The cleared lysate was transferred to a fresh tube for further analysis.

### Harvesting and concentration of culture medium

All medium samples analyzed by sodium dodecyl sulfate-polyacrylamide gel electrophoresis (SDS-PAGE) and/or immunoblot were harvested by centrifugation at 10,000×*g*, 4 °C for 20 min and subsequently filtered with 0.45 µm PES membrane filters. Samples were then flash-frozen in liquid N_2_, lyophilized and stored at − 80 °C until further analysis.

### Preparation of periplasm, total membrane and thylakoid enriched fractions

The periplasmic fraction of cultures was extracted following a previously described protocol [37] from 50 mL of an OD_750 nm_ ≈ 4 culture. The pellet fraction (i.e. spheroplasts) was subjected to further fractionation. Spheroplasts were lysed in lysis buffer without Triton X-100 and broken as described previously (“Preparation of cleared cell lysates” section). Debris, beads and unbroken cells were collected by centrifugation at 3000×*g* for 5 min at 4 °C. The subsequent fractionation is based on a previously described protocol [38]. Briefly, the lysed spheroplasts were centrifuged at 100,000×*g*, 4 °C for 60 min using a MLS-50 swinging-bucket rotor (Beckman Coulter). The supernatant (containing the cytosolic fraction) was collected in a separate tube and the membrane fraction was resuspended in lysis buffer. An aliquot of the total membrane fraction was kept for further analysis, the rest was loaded on a two-phase sucrose gradient [30% (w/v) and 50% (w/v)] for enrichment of the thylakoid membranes. The sucrose gradient was centrifuged for 60 min at 4 °C and 150,000×*g*. The thylakoid membranes were collected from the 30 to 50% sucrose interphase.

### Sample preparation for SDS-PAGE, Coomassie and Oriole staining

Protein content of samples for SDS-PAGE was analyzed using a Pierce 660 nm protein assay reagent (Thermo Scientific) following the manufacturer’s recommendations with a standard curve from 0 to 2000 µg mL^−1^ bovine serum albumin. Samples were incubated for 5 min at 95 °C with sample loading buffer [containing 2% SDS and 0.1 M dithiothreitol (DTT)] prior to separation by SDS-PAGE. Proteins were separated on 12% Criterion XT bis–tris pre-cast gels (Bio-Rad Laboratories) in 2-morpholinoethanesulphonic acid (MES) running buffer at 180 V. Afterwards, the gels were either stained with Coomassie Blue, Oriole Fluorescent Gel Stain (Bio-Rad Laboratories) (protocol according to manufacturer’s instructions) or immunoblotted (see “Immunoblot analysis”).

### Immunoblot analysis

After SDS-PAGE separation, proteins were transferred onto a 0.2 µm polyvinylidene difluoride (PVDF) membrane using a Trans-Blot^®^ Turbo transfer system (Bio-Rad Laboratories) for 7 min at 25 V. Membranes were blocked with 5% skimmed milk powder (w/v) in phosphate buffered saline with 0.05% Tween-20 (PBS-T) buffer for 60 min at room temperature and incubated in a primary antibody solution at 4 °C overnight. Various primary antibodies were used with the following dilutions: Anti-HA 1:1000 (Sigma-Aldrich), Anti-RbcL 1:5000 (Agrisera) and Anti-PsbA 1:8000 (Agrisera). The membrane was then washed with PBS-T and incubated for 60 min at room temperature with an Anti-Rabbit horseradish peroxidase (HRP)-conjugated secondary antibody (Dako) at a dilution of 1:5000. After more washing steps, the HRP signal was developed using SuperSignal West Dura Substrate (Thermo Scientific) and detected with a ChemiDoc MP imaging system (Bio-Rad Laboratories).

### Mass spectrometry analysis

Samples for mass spectrometry were separated by SDS-PAGE and stained with Coomassie Blue. The band of interest was excised from the gel and transferred to a de-staining solution of 50% ethanol (EtOH) and 25 mM NH_4_HCO_3_. In-gel samples were then prepared for nano-liquid chromatography/mass spectrometry/mass spectrometry (LC/MS/MS) analysis as previously described [39]. Briefly, gel samples were reduced and cysteines alkylated with tris(2-carboxyethyl)phosphine and chloroacetamide, respectively. Peptides were generated via sequential digestion of protease lys-C and trypsin. Peptide containing samples were desalted using C_18_ reversed phase material prior to LC/MS/MS analysis. Proteomics analysis was performed on a Orbitrap Q-Exactive HF-X mass spectrometer (Thermo Scientific) coupled to EASY-nLC 1200 system. Peptides were injected onto an in-house packed nano-column (75 µM internal diameter and 15 cm length) packed with 1.9 µM C_18_ beads (Dr. Maisch GmbH). EASY-nLC 1200 was operated at 250 nL min^−1^ with an increasing gradient to 25% buffer B (80% acetonitrile, 0.1% formic acid). Gradient was increased to 40% in 5 min followed by a quick increase to 80% B where it was held for 2 min. Column was re-equilibrated to 5% where it was held for 5 min. The mass spectrometer was operated in positive mode using a Top15 data dependent acquisition (DDA) method. MS resolution was set to 120,000 and MS/MS fragment resolution was set to 30,000. Precursors were fragmented at a normalized collision energy (NCE) of 28 and precursors with same mass were excluded from fragmentation for 30 s thereafter. Raw data were analyzed using MaxQuant software with Andromeda search engine [40]. Peptides and proteins were searched against the reference proteome of *S. elongatus* PCC 7942 with the added *Tf*AA10A protein FASTA sequences.

### Densitometry

For densitometry, cultures were grown to an OD_750 nm_ = 25 and induced with 1 mM IPTG for 48 h. 10 mL of cleared culture medium (OD_750 nm_ = 28) were harvested, lyophilized and resuspended in 250 µL of 10 mM Tris–HCl buffer (pH 7.5). Five microlitre of sample were analyzed by SDS-PAGE and subsequent Oriole (Bio-Rad Laboratories) staining together with a standard curve of 50 ng, 100 ng, 150 ng and 200 ng of a commercial *Tf*LPMO10A (NZYTech). Protein quantities were estimated using densitometric analysis with Image Lab 6.0 Software (Bio-Rad Laboratories).

### Growth analysis

Growth was analyzed in a Multicultivator MC1000 (Photon Systems Instruments) in glass tubes bubbled with 3% CO_2_ supplemented air at 38 °C. The cultures were illuminated with a linear light gradient from 250 µmol photons m^−2^ s^−1^ to 900 µmol photons m^−2^ s^−1^ over 12 h, followed by continuous illumination with 900 µmol photons m^−2^ s^−1^. Pre-cultures were grown in the same conditions. The experimental cultures were inoculated, in triplicate, to a cell density of 1 × 10^8^ cells mL^−1^ from pre-cultures and directly induced with 1 mM IPTG at timepoint 0 h. During the growth experiment, aliquots were regularly taken from the cultures to measure OD at 750 nm with a spectrophotometer, count cells using a Muse^®^ Cell Analyzer (Merck MilliporeSigma) and to extract pigments. OD samples were diluted to OD < 1 before the measurement. Pigment content (chlorophyll and carotenoids) was estimated as described elsewhere [41] with slight modifications of the extraction protocol (cultures were centrifuged at 20,000×*g* for 10 min at 4 °C prior to and after pigment extraction). Each cell count was performed in technical triplicates and the average of these calculated. Averages of the biological replicates were calculated and the standard error is displayed (n = 3). For OD measurements and pigment extraction, one technical replicate was analyzed for all biological replicates at each shown timepoint. Means were compared using a one-way ANOVA performed in Prism (version 8.0, GraphPad Software).

### *Tf*AA10A purification and copper reconstitution

*Tf*AA10A was purified from the culture medium of a 1 L late log phase culture. Cells were harvested at 10,000×*g*, 4 °C for 20 min. Complete protease inhibitor cocktail (Roche) was added to the cell-free medium (1 tablet per 100 mL) and, subsequently, the medium was filtered with a 0.45 µm PES membrane filter. The medium was loaded onto a 10-mL Q-Sepharose column (Q-Sepharose Fast Flow, GE Healthcare) which was washed in 20 mM HEPES pH 7.2, 5% glycerol, 1 mM MgCl_2_ and 5 mM DTT. DTT is a known LPMO reducing agent, therefore, when possible, should be avoided to prevent protein damage during purification. *Tf*AA10A was eluted using two column volumes of the same buffer containing 150 and 200 mM NaCl. All elution fractions were concentrated using Vivaspin 20 centrifugal concentrators with a 10 kDa molecular weight cut-off (GE Healthcare) and subjected to affinity chromatography using 100 µL Pierce Anti-HA Agarose resin (Thermo Scientific) according to the manufacturer’s protocol. *Tf*AA10A was eluted with 10 mM Tris–HCl buffer (pH 7.5) and 3.5 M MgCl_2_, and desalted with Micro Bio-Spin 6 columns pre-hydrated with 10 mM Tris–HCl buffer (pH 7.5) (Bio-Rad Laboratories). Purity was confirmed by SDS-PAGE and immunoblotting. Copper reconstitution of the purified *Tf*AA10A was done by incubating the protein with CuCl_2_, in a 1:2 molar ratio (*Tf*AA10A:CuCl_2_) at 4 °C overnight. Excess CuCl_2_ buffer was removed with Micro Bio-Spin 6 desalting columns pre-hydrated with 10 mM Tris–HCl buffer (pH 7.5) (Bio-Rad Laboratories). The reconstituted protein was then used for the activity assays described in the next sections.

### Amplex red assay

The Amplex red assay [42] was performed on a SpectraMax M2 multi-detection microplate reader using a black 96-well plate with clear bottom (Thermo Scientific). All the reactions were performed in 20 mM sodium phosphate buffer, pH 6.0 at 37 °C. 200 µL of reaction mixture contained 100 µM EDTA (10 mM stock), 80 µM ascorbate (1 mM stock), 50 µM Ampliflu red (5 mM stock), 20 U mL^−1^ HRP (300 U mL^−1^ stock) and 1 µg purified and reconstituted *Tf*AA10A. The negative control omitted the enzyme. The buffer control contained only 20 mM sodium phosphate buffer, pH 6.0. Resorufin fluorescence was recorded with an excitation and emission wavelength of 557 nm and 583 nm, respectively. Fluorescence was measured from the bottom for 10 min with 5 s initial shaking. Averages of the replicates were calculated and the standard error is displayed (n = 3). Means were compared using a one-way ANOVA performed in Prism (version 8.0, GraphPad Software).

### LPMO catalyzed oxidation of PASC

Phosphoric acid swollen cellulose (PASC) substrate was prepared by treating Avicel (Merck MilliporeSigma), a crystalline cellulose, with phosphoric acid as previously described [43]. Briefly, 4 g of Avicel was dispersed into 100 mL of phosphoric acid (86% w/v) at 60 °C and magnetically stirred for an hour. 1900 mL H_2_O was slowly dripped into the solution while stirring. The suspension was then incubated at 4 °C for sedimentation. After sedimentation, the supernatant was removed, the suspension was washed four times with H_2_O and the pH was neutralized using Na_2_CO_3_. *Tf*AA10A catalyzed oxidation of PASC was performed in 50 mM phosphate buffer (pH 7.5). All reaction mixtures contained 2 mM ascorbate, 0.75% w/v PASC and 2 µg purified and reconstituted *Tf*AA10A. The negative control omitted the enzyme. Reactions were carried out at 50 °C for 24 h. Reactions were stopped by boiling the samples in the presence of 1 mM EDTA. Samples were then filtered with a 0.45 µM PES membrane filter and analyzed by high-performance anion exchange chromatography (HPAEC). HPAEC analysis of the released oligosaccharides from the cellulosic substrate was performed on an ICS-5000+ system, equipped with a PAD detector (Thermo Scientific), with a CarboPac PA1 column (two 2 × 50 mm guard columns followed by a 2 × 250 mm analytical column), and operated at 0.25 mL min^−1^ and 30 °C. The chromatographic separation of aldonic acids was carried out as previously described [44]. For the elution the following gradient was applied (Eluent A: 0.1 M NaOH; Eluent B: 1 M NaOAc in 0.1 M NaOH): 100% A:0% B to 90% A:10% B (10 min), then to 83.1% A:16.9% B (25 min) and lastly 0% A:100% B (30 min). For reconditioning of the column 100% A:0% B was applied for 15 min (35–50 min). The cello-oligosaccharide peaks were assigned according to elution profiles of synthesized oxidized oligosaccharide standards (DP2_ox_–DP6_ox_), commercially available native oligosaccharides (DP3–DP6) and based on inferences from previous studies [45, 46]. The oxidized oligosaccharide standards (DP2_ox_–DP6_ox_) were synthesized by the iodine oxidation method modified from previous studies [47, 48].

## Results

### Generation of the *S. elongatus* UTEX 2973 strains “*Tf*AA10A” and “TorA-*Tf*AA10A”

**Fig. 1:**
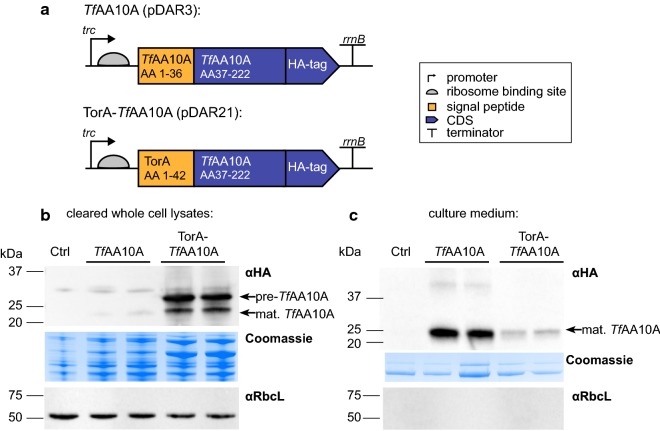
Overview of *Tf*AA10A expression in *Synechococcus elongatus* UTEX 2973. **a** Overview of *Tf*AA10A expression cassettes inserted into the pDF-trc self-replicating plasmid used for *S. elongatus* UTEX 2973 transformation. *Tf*AA10A encodes the native protein sequence. For TorA-*Tf*AA10A the N-terminal native signal peptide was removed and replaced by the *E. coli* TorA signal peptide. In both constructs, a C-terminal human influenza hemagglutinin (HA) epitope tag was added to the protein for detection purposes. **b** Cellular lysates of two independent transformants expressing *Tf*AA10A and TorA-*Tf*AA10A detected by immunoblot analysis using an HA antibody (αHA). Pre- and mature (processed) protein bands are indicated with arrows. Ctrl indicates an empty pDF-trc vector control strain. Lanes are normalized to 10 µg of total protein. A Coomassie-stained polyacrylamide gel is shown as a loading control. An αRbcL blot is shown as a cell lysis control. **c** Cell-free culture medium of two independent transformants expressing *Tf*AA10A and TorA-*Tf*AA10A detected by immunoblot analysis using αHA. The mature (processed) protein band is indicated with an arrow. Ctrl indicates an empty vector control strain. Lanes are normalized to 2 mL of cell-free culture medium. A Coomassie-stained polyacrylamide gel is shown as a loading control. An αRbcL blot is shown as a cell lysis control

*Thermobifida fusca* is a model cellulolytic bacterium which secretes two LPMOs: the 22.7 kDa single-domain *Tf*AA10A (formerly E7) and the 44.7 kDa three-domain *Tf*AA10B (formerly E8) [30]. The activity of *Tf*AA10A is well characterized and its X-ray structure has been described. It is commercially available and is now considered a model enzyme for cellulose degradation in bacteria [45, 49, 50]. Therefore, for this study, we chose the *T. fusca* LPMO AA10A (*Tf*AA10A) for expression in *S. elongatus* UTEX 2973. A custom-synthesized and codon-optimized DNA sequence encoding the full length *Tf*AA10A protein fused to a C-terminal HA epitope tag was inserted into the multiple cloning site of the self-replicating broad-host-range pDF-trc expression vector [51]. In the absence of a *Tf*AA10A specific antibody, the HA epitope tag allowed for detection of the protein. The generated plasmid was termed pDAR3. Additionally, a second construct was generated where the DNA sequence encoding the native *Tf*AA10A N-terminal Sec signal peptide (AA residues 1–36) was replaced with the N-terminal signal peptide of the *E. coli* trimethylamine *N*-oxide reductase (TorA) which is a known model Tat pathway signal peptide. This second plasmid was named pDAR21 (Fig. 1a). To generate the modified cyanobacterial strains, the plasmids pDAR3, pDAR21 and pDF-trc [control plasmid without a gene of interest (GOI)] were conjugated into *S. elongatus* UTEX 2973 using biparental mating with an *E. coli* helper strain. The generated strains were selected on spectinomycin-containing growth medium. Single colonies (up to 5 per construct) were analyzed by PCR for the presence of the GOI (Additional file 1: Figure S1) to confirm successful transformation. The generated *S. elongatus* UTEX 2973 strains were named *Tf*AA10A (transformed with pDAR3 plasmid) and TorA-*Tf*AA10A (transformed with pDAR21 plasmid) (Fig. 1a). The *S. elongatus* UTEX 2973 strain containing the pDF-trc plasmid without a GOI will be referred to as “Ctrl”.

### Expression of *Tf*AA10A in *S. elongatus* UTEX 2973

Two independent colonies from each transformant, confirmed to contain the respective GOI, were subjected to immunoblot analysis to screen for expression of the *Tf*AA10A protein (Fig. 1b, c). To determine the location of *Tf*AA10A, the cleared cellular lysates and cell-free culture medium were analyzed separately with an anti-HA antibody (Fig. 1b, c). The expected size of the mature *Tf*AA10A, together with the C-terminal HA tag, is 22.4 kDa. In the cellular lysates of the *Tf*AA10A strain, only trace amounts of mature protein were found and the pre-protein could not be detected. In contrast, in the TorA-*Tf*AA10A strain, both the pre-protein and the mature form were found in the cellular lysate (Fig. 1b). In the culture medium of the *Tf*AA10A strain, the mature protein was found at the expected size, however, only a small amount of mature protein was detected in the medium of the TorA-*Tf*AA10A strain (Fig. 1c). To verify if the presence of the protein in the medium of both strains was due to active secretion rather than cell lysis, medium samples were also immunoblotted with an anti-RbcL antibody. The large subunit of ribulose-1,5-bisphosphate carboxylase/oxygenase (RbcL) could only be detected in the cellular lysates (Fig. 1b) whereas no protein was found in the medium (Fig. 1c). These results suggested that the LPMO was efficiently translocated across both membranes in the *Tf*AA10A strain but that this was not the case in the TorA-*Tf*AA10A strain.

### Unprocessed *Tf*AA10A accumulates in the plasma membrane of the TorA-*Tf*AA10A strain

**Fig. 2:**
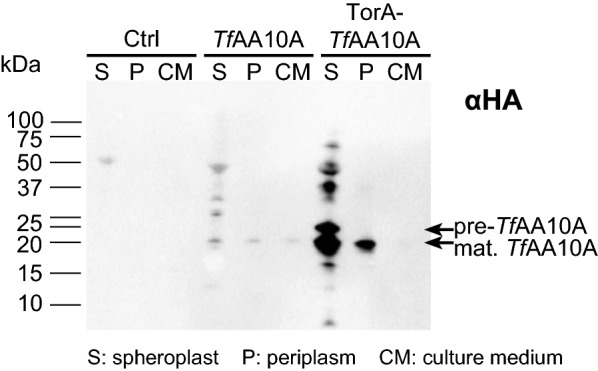
*Tf*AA10A and TorA-*Tf*AA10A location study in *S. elongatus* UTEX 2973 transformants. Proteins detected by immunoblot analysis using an HA antibody (αHA). Pre- and mature protein bands are indicated with arrows. Ctrl indicates an empty vector control strain. Protein loaded per lane was normalized to an equivalent of 1 × 10^8^ cells. *S* spheroplasts, *P* periplasm, *CM* culture medium

To determine where the *Tf*AA10A protein was accumulating within the cell, cultures of each strain were fractionated into spheroplasts and periplasm (Fig. 2). Confirming what was previously observed (Fig. 1b, c), very little protein accumulated intracellularly in the *Tf*AA10A strain. However, in the TorA-*Tf*AA10A strain, both the pre-protein and mature *Tf*AA10A were found in large amounts in the spheroplasts and some processed (mature) *Tf*AA10A was detected in the periplasmic fraction (Fig. 2).

**Fig. 3:**
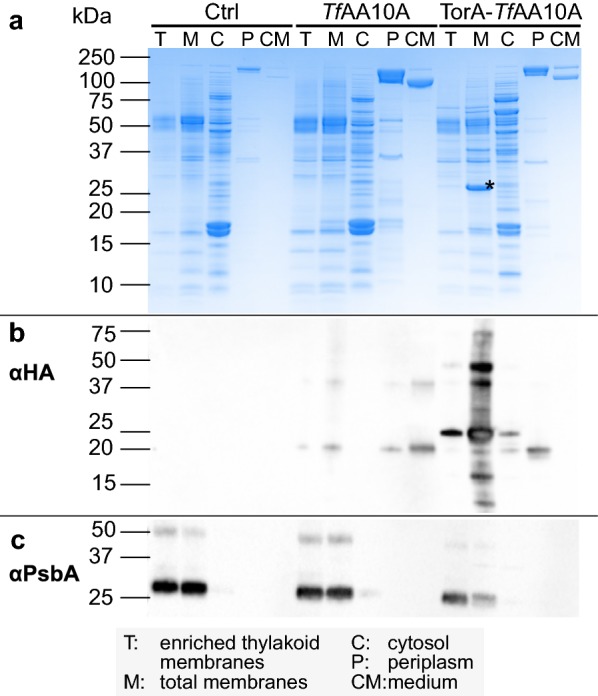
Fractionation of *Synechococcus elongatus* UTEX 2973 strains expressing *Tf*AA10A and TorA-*Tf*AA10A. **a** Coomassie-stained polyacrylamide gel. The TorA-*Tf*AA10A pre-protein band is indicated with an asterisk. Lanes are normalized to 5 µg of total protein. **b** αHA immunoblot. Lanes are normalized to 1 µg of total protein. **c** αPsbA immunoblot. Lanes are normalized to 5 µg of total protein. CM lanes in all panels are normalized to 25 µL of cell-free medium. Ctrl indicates an empty vector control strain

To determine the subcellular localization of *Tf*AA10A protein accumulation more precisely, spheroplasts were further fractionated into cytosol (soluble fraction), total membranes/insoluble fraction and thylakoid enriched membranes (Fig. 3). Separation of the individual fractions by SDS-PAGE showed that the most prominent band found in the total membrane fraction of the TorA-*Tf*AA10A strain, matched the predicted size of unprocessed TorA-*Tf*AA10A (26.8 kDa) (Fig. 3a, marked with an asterisk). The band was much less abundant in the thylakoid enriched fraction of TorA-*Tf*AA10A suggesting that the majority of the protein is localized to the plasma membrane (Fig. 3a, b). The identity of the band was confirmed as unprocessed TorA-*Tf*AA10A by fingerprint mass spectrometry (Additional file 1: Figure S2). Herewith, it became apparent that the TorA-*Tf*AA10A strain was unable to efficiently secrete *Tf*AA10A. However, in the *Tf*AA10A strain we observed only small amounts of the LPMO in the membrane fractions and the periplasm suggesting more efficient secretion of the protein (Fig. 3b). Therefore, we proceeded to characterize the secretion ability of the *Tf*AA10A strain in more detail.

### *Tf*AA10A is efficiently secreted with its native Sec signal peptide

To assess correct processing of the signal peptide and secretion yields, the protein from the *Tf*AA10A strain was harvested from the culture medium and concentrated for further analysis. SDS-PAGE analysis of the medium suggested that the secreted *Tf*AA10A was processed correctly as the observed protein size matched the expected size of the mature protein (Figs. 1c, 2, 3). To further confirm this observation, the protein was analyzed by cutting out the band from an SDS-PAGE gel and subsequent mass spectrometry fingerprint analysis (Additional file 1: Figure S3). The mature N-terminal tryptic peptide was identified and furthermore, no evidence of the *Tf*AA10A signal peptide was found. This suggests the secreted protein was efficiently processed. We then proceeded to determine secretion yields by densitometric analysis utilizing a fluorescent gel stain and a commercially available *Tf*AA10A of known concentration for a standard curve (Additional file 1: Figure S4). Based on the densitometric analysis of absolute protein abundance, protein yields were estimated to be 779 ± 40 µg L^−1^.

### The *S. elongatus* UTEX 2973 secretes a catalytically active *Tf*AA10A

**Fig. 4:**
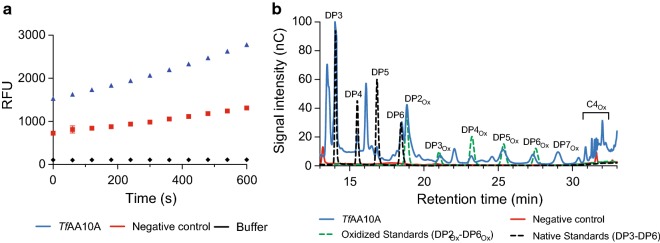
*S. elongatus* UTEX 2973 *Tf*AA10A activity assays. **a** Amplex red assay. The assay was performed in 20 mM sodium phosphate buffer, pH 6.0. All reaction mixtures contained 100 µM EDTA, 80 µM ascorbate, 50 µM Ampliflu red, 20 U mL^−1^ HRP and 1 µg purified and reconstituted *Tf*AA10A. The negative control omitted the enzyme. The buffer control sample contained only 20 mM sodium phosphate buffer, pH 6.0. Resorufin fluorescence was recorded with an excitation and emission wavelength of 557 nm and 583 nm, respectively. All experiments were carried out at 37 °C. Results are an average of three replicates and error bars represent the standard deviation. Nonvisible error bars are smaller than the data symbol. **b** PASC oxidation by *Tf*AA10A. *Tf*AA10A catalyzed oxidation of PASC was performed in 50 mM phosphate buffer (pH 7.5). All reaction mixtures contained 2 mM ascorbate, 0.75% w/v PASC and 2 µg purified and reconstituted *Tf*AA10A. The negative control omitted the enzyme. Reactions were carried out at 50 °C for 24 h. Peak assignments are based on cello-oligosaccharide standards and inferences from previous studies [45, 46]. DP—degree of polymerization. DP3–DP6—commercially available native oligosaccharide standards. DP2_ox_–DP6_ox_—synthesized oxidized oligosaccharide standards. DP3—cellotriose, DP4—cellotetraose, DP5—cellopentaose and DP6—cellohexaose, DP2_ox_—cellobionic acid, DP3_ox_—cellotrionic acid, DP4_ox_—cellotetraonic acid, DP5_ox_—cellopentaonic acid, DP6_ox_—cellohexaonic acid and DP7_ox_—celloeptaonic acid. Results are an average of three replicates. Full chromatogram can be found in Additional file 1: Figure S5

To determine if the secreted protein was active, the purified and copper reconstituted *Tf*AA10A was initially tested for activity using the Amplex red assay. This coupled assay measures the generation of hydrogen peroxide, a futile oxygen-reducing reaction of the LPMO in the absence of a substrate [42]. Samples of the cyanobacterial *Tf*AA10A showed a significantly stronger increase in fluorescence intensity compared to the control samples (*p* < 0.001) (Fig. 4a). Then, to confirm that the cyanobacterial *Tf*AA10A was catalytically active, we incubated the protein with the model substrate PASC and ascorbic acid and measured the released oligosaccharides by high-performance anion exchange chromatography (HPAEC) (Fig. 4b). The chromatograms obtained from the enzymatic assay were compared to a set of synthesized oxidized oligosaccharide standards (DP2_ox_–DP6_ox_) and commercially available non-oxidized (i.e. native) oligosaccharides (DP3–DP6). The oxidized peaks, and native counterparts, matched a substrate oxidation pattern typical for C1-oxidizing LPMOs (Fig. 4b).

### *S. elongatus* UTEX 2973 *Tf*AA10A strain has longer lag phase but shows overall no significant growth deficit

**Fig. 5:**
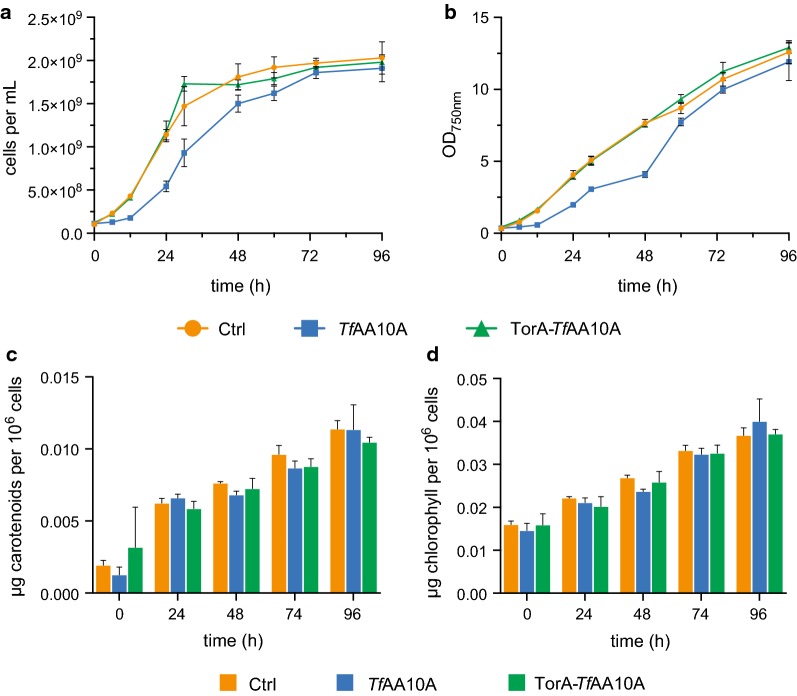
Biomass and pigment accumulation of *Synechococcus elongatus* UTEX 2973 over 96 h. *S. elongatus* UTEX 2973 transformants were grown in modified P4-TES CPH medium at 38 °C and bubbled with 3% CO_2_ supplemented air. The cultures were illuminated with a linear light gradient from 250 µmol photons m^−2^ s^−1^ to 900 µmol photons m^−2^ s^−1^ over 12 h, followed by continuous illumination with 900 µmol photons m^−2^ s^−1^. Cell number (**a**), optical density (750 nm) (**b**), carotenoid (**c**) and chlorophyll content (**d**) were determined. Carotenoid and chlorophyll concentrations are normalized to 1 × 10^6^ cells. Error bars represent the standard deviation (n = 3)

As both *Tf*AA10A and TorA-*Tf*AA10A strains successfully expressed the heterologous *Tf*AA10A, we wanted to determine the metabolic impact of heterologous LPMO expression on the cyanobacterial transformants. Therefore, we analyzed growth (cell numbers and optical density) and pigment content (chlorophyll and carotenoids), over the course of 96 h, in the two LPMO strains and an empty vector control strain (Fig. 5). In the initial 12 h of the experiment, the *Tf*AA10A strain presented slower growth and, consequently, lower cell numbers throughout the experiment, however, after 72 h there was no significant difference between the three strains (*p* = 0.722) (Fig. 5a, b). Chlorophyll and carotenoid values, normalized to cell number, showed that there were no significant differences in chlorophyll (*p* = 0.947) or carotenoid (*p* = 0.915) amounts across the three strains (Fig. 5c, d).

## Discussion

In this study, we have successfully expressed and secreted the *T. fusca* LPMO *Tf*AA10A in the fast-growing cyanobacterium *S. elongatus* UTEX 2973. Two different strains were generated for this study—one strain expressing the native *Tf*AA10A sequence with its N-terminal Sec signal peptide and a second strain which has the native N-terminal signal peptide replaced by the TorA signal peptide, a model signal peptide for Tat translocation studies. The native *Tf*AA10A was efficiently secreted to the medium with virtually no protein found in the cellular lysates. However, the Tat-targeted TorA-*Tf*AA10A was mostly found unprocessed, and degraded, in the plasma membrane.

### Secretion via the Tat pathway in *S. elongatus* UTEX 2973 needs further improvement

The Tat pathway has the particularity of translocating fully folded proteins with an inbuilt “proof-reading” system capable of rejecting misfolded or cofactor-lacking substrates [52–54]. In bacteria, the Tat pathway is mainly used for the translocation of complex cofactor-containing redox enzymes into the periplasm and the extracellular space [55, 56]. Therefore, we hypothesized that Tat-mediated secretion could potentially be advantageous for the copper cofactor-containing *Tf*AA10A. In Fig. 3 we show that the majority of the protein is correctly targeted to the plasma membrane and to the periplasm (rather than into the thylakoid lumen). This is in line with previous reports that show TorA-GFP expression in the model cyanobacterium *Synechocystis* PCC 6803 led to GFP accumulation in the periplasm rather than the thylakoid lumen [57]. However, TorA-*Tf*AA10A mostly accumulated unprocessed in the plasma membrane with only a minor fraction being translocated to the periplasm. While the exact reason for this is not clear, we can speculate that if the protein cannot fold correctly in the cytoplasmic environment, or if the copper cofactor is not adequately incorporated, it may not be translocated by the Tat machinery. As we have only tested the TorA signal peptide, we cannot rule out that our observations are specific to this signal peptide in *S. elongatus* UTEX 2973. Other Tat-targeting signal peptides may be able to improve secretion with the cyanobacterial Tat system.

### Type IV pilus (T4P) system may be involved in translocation across the outer membrane in *S. elongatus* UTEX 2973

In the Gram-positive bacterium *T. fusca*, *Tf*AA10A is secreted unfolded through the Sec pathway. Due to the single membrane architecture of this bacterium, the protein is translocated directly to the extracellular environment [30]. Therefore, we were interested in testing if the Gram-negative cyanobacterial secretion system could recognize and secrete a heterologous protein of Gram-positive origin. Our initial results demonstrated that *Tf*AA10A secretion was indeed extremely efficient and virtually all the protein was translocated into the culture medium. Translocation across the plasma membrane, i.e. export to the periplasm, is likely to be mediated by the Sec pathway. The Sec pathway is conserved across the bacterial kingdom [58] and both *T. fusca* and *S. elongatus* UTEX 2973 have a complete set of genes encoding the different Sec components [59, 60]. However, we can only speculate on the precise manner through which the protein is sorted in the periplasm and translocated across the outer membrane. Secretion in cyanobacteria is poorly characterized, however, two potential routes are known: a one-step, Sec or Tat independent, cytoplasm-to-medium route and a two-step periplasmic route that relies on Sec or Tat to mediate translocation across the plasma membrane and requires the engagement of additional components for translocation across the outer membrane [23]. Genome-wide bioinformatics analysis have identified both secretion systems in *S. elongatus* PCC 7942: the one-step type I secretion system (T1SS) and the two-step type IV pilus (T4P) assembly system [61]. Apart from a point mutation in the T4P protein PilN, the T4P system in *S. elongatus* UTEX 2973 seems to be identical to *S. elongatus* PCC 7942 [9]. Therefore, the presence of Sec or Tat targeting signal peptides in our different constructs suggest that secretion is more likely to be mediated by the two-step T4P cyanobacterial secretion system.

### Efficient Sec-mediated secretion in *S. elongatus* UTEX 2973 is promising for biotechnological applications

Precise cleavage of LPMO signal peptides is of utmost importance due to the fact that the N-terminal histidine of the mature protein coordinates the active site copper ion [26]. Therefore, it was crucial to demonstrate that the secreted enzyme was catalytically active. The secreted *Tf*AA10A was first tested with an Amplex red assay which suggested the enzyme was active. We proceeded to test the activity of *Tf*AA10A on the model LPMO substrate PASC. The activity chromatogram showed a clear C1 oxidation profile leading us to conclude that the signal peptide was processed correctly during secretion. To achieve high-titer secretion, we took advantage of the fast-growth of *S. elongatus* UTEX 2973 under high-light and elevated CO_2_ levels. After 5 days of growth an OD_750_ of 25 was achieved and the culture was induced. Culture induction was done in the stationary phase because existing secretion literature supports the hypothesis that a slower translation rate can improve protein folding and translocation yields [62]. After harvesting, densitometric analysis indicated a *Tf*AA10A yield of 779 ± 40 µg L^−1^ in the culture medium without further optimization (Additional file 1: Figure S4). Although the lack of quantification data in other cyanobacterial secretion studies hinders widespread comparisons, to our knowledge, this is the highest yield reported to date for the secretion of a heterologous enzyme in cyanobacteria [24, 25, 63]. Although our reported yield of secreted LPMO is approximately 25% of previously reported yields for *E. coli Tf*AA10A expression (3 mg L^−1^), the majority of *Tf*AA10A in *E. coli* was exported to the periplasm [30], whereas the *S. elongatus* UTEX 2973 *Tf*AA10A was fully secreted to the medium. The combination of high expression and low impact on cell growth and final biomass levels suggests promising potential for further optimization and biotechnological exploitation. Previous studies have suggested that cyanobacterial secretion of heterologous proteins may have applications in microcystin degradation [64], heavy metal bioremediation [65] and CO_2_ sequestration [63]. We believe this can also be extended to whole-cell biocatalysis where cyanobacteria have the potential to become light-driven cell factories capable of secreting active enzymes to sustain extracellular biotransformations.

## Conclusion

We have demonstrated for the first time that *S. elongatus* UTEX 2973 is capable of high-titer secretion of a heterologous and industrially relevant enzyme. Although *S. elongatus* UTEX 2973 is a relatively new organism to be characterized for biotechnological applications, it is likely to become a popular option among cyanobacteria due to its properties of fast-growth, environmental resistance and genetic tractability. Additionally, by demonstrating that protein secretion can be achieved with a Gram-positive signal peptide, we have dramatically expanded the library of potential signal peptides for cyanobacterial applications. Establishing robust systems of heterologous enzyme secretion in cyanobacteria is an important step towards the development of light-driven applications in bioremediation and whole-cell biocatalysis.

## Additional file

**Additional file 1: Figure S1.** PCR analysis of *S. elongatus* UTEX 2973 strains *Tf*AA10A (pDAR3) and TorA-*Tf*AA10A (pDAR21). **Figure S2.** Identification of the TorA signal peptide in the pre-protein TorA-*Tf*AA10A, found in the plasma membrane fraction, by mass spectrometry. **Figure S3.** Identification of the secreted *Tf*AA10A by mass spectrometry. **Figure S4.** Densitometric analysis of secreted *Tf*AA10A. **Figure S5.** PASC oxidation by *Tf*AA10A.

## References

[1] Klemenčič M, Nielsen AZ, Sakuragi Y, Frigaard N-U, Čelešnik H, Jensen PE, Gonzalez-Fernandez C, Muñoz R (2017). Synthetic biology of cyanobacteria for production of biofuels and high-value products. Microalgae-based biofuels and bioproducts.

[2] Ko SC, Lee HJ, Choi SY, Choi J, Woo HM (2018). Bio-solar cell factories for photosynthetic isoprenoids production. Planta.

[3] Noreña-Caro D, Benton MG (2018). Cyanobacteria as photoautotrophic biofactories of high-value chemicals. J CO2 Util.

[4] Knoot CJ, Ungerer J, Wangikar PP, Pakrasi HB (2018). Cyanobacteria: promising biocatalysts for sustainable chemical production. J Biol Chem.

[5] Lin P-C, Pakrasi HB (2018). Engineering cyanobacteria for production of terpenoids. Planta.

[6] Nielsen AZ, Mellor SB, Vavitsas K, Wlodarczyk AJ, Gnanasekaran T, de Jesus MPRH (2016). Extending the biosynthetic repertoires of cyanobacteria and chloroplasts. Plant J.

[7] Hagemann M, Hess WR (2018). Systems and synthetic biology for the biotechnological application of cyanobacteria. Curr Opin Biotechnol.

[8] Jaiswal D, Sengupta A, Sohoni S, Sengupta S, Phadnavis AG, Pakrasi HB (2018). Genome features and biochemical characteristics of a robust, fast growing and naturally transformable cyanobacterium *Synechococcus elongatus* PCC 11801 isolated from India. Sci Rep.

[9] Yu J, Liberton M, Cliften PF, Head RD, Jacobs JM, Smith RD (2015). *Synechococcus elongatus* UTEX 2973, a fast growing cyanobacterial chassis for biosynthesis using light and CO_2_. Sci Rep.

[10] Ungerer J, Wendt KE, Hendry JI, Maranas CD, Pakrasi HB (2018). Comparative genomics reveals the molecular determinants of rapid growth of the cyanobacterium *Synechococcus elongatus* UTEX 2973. Proc Natl Acad Sci USA.

[11] Ungerer J, Lin P-C, Chen H-Y, Pakrasi HB (2018). Adjustments to photosystem stoichiometry and electron transfer proteins are key to the remarkably fast growth of the cyanobacterium *Synechococcus elongatus* UTEX 2973. mBio.

[12] Song K, Tan X, Liang Y, Lu X (2016). The potential of *Synechococcus elongatus* UTEX 2973 for sugar feedstock production. Appl Microbiol Biotechnol.

[13] Hendry JI, Gopalakrishnan S, Ungerer J, Pakrasi HB, Tang Y, Maranas CD (2019). Genome-scale fluxome of *Synechococcus elongatus* UTEX 2973 using transient 13C-labeling data. Plant Physiol.

[14] Tan X, Hou S, Song K, Georg J, Klähn S, Lu X (2018). The primary transcriptome of the fast-growing cyanobacterium *Synechococcus elongatus* UTEX 2973. Biotechnol Biofuels.

[15] Ungerer J, Pakrasi HB (2016). Cpf1 is a versatile tool for CRISPR genome editing across diverse species of cyanobacteria. Sci Rep.

[16] Li S, Sun T, Xu C, Chen L, Zhang W (2018). Development and optimization of genetic toolboxes for a fast-growing cyanobacterium *Synechococcus elongatus* UTEX 2973. Metab Eng.

[17] Cengic I, Uhlén M, Hudson EP (2018). Surface display of small affinity proteins on *Synechocystis* sp. strain PCC 6803 mediated by fusion to the major type IV Pilin PilA1. J Bacteriol.

[18] Chungjatupornchai W, Kamlangdee A, Fa-aroonsawat S (2011). Display of organophosphorus hydrolase on the cyanobacterial cell surface using Synechococcus outer membrane protein A as an anchoring motif. Appl Biochem Biotechnol.

[19] Fedeson DT, Ducat DC (2017). Cyanobacterial surface display system mediates engineered interspecies and abiotic binding. ACS Synth Biol.

[20] Ferri S, Nakamura M, Ito A, Nakajima M, Abe K, Kojima K (2015). Efficient surface-display of autotransporter proteins in cyanobacteria. Algal Res.

[21] Frain KM, Gangl D, Jones A, Zedler JAZ, Robinson C (2016). Protein translocation and thylakoid biogenesis in cyanobacteria. Biochim Biophys Acta Bioenergy.

[22] Desvaux M, Hébraud M, Talon R, Henderson IR (2009). Secretion and subcellular localizations of bacterial proteins: a semantic awareness issue. Trends Microbiol.

[23] Gonçalves CF, Lima S, Tamagnini P, Oliveira P, Mishra AK, Tiwari DN, Rai AN (2019). Cyanobacterial secretion systems: understanding fundamental mechanisms toward technological applications. Cyanobacteria.

[24] Sergeyenko TV, Los DA (2003). Cyanobacterial leader peptides for protein secretion. FEMS Microbiol Lett.

[25] Lima S, Oliveira P, Tamagnini P (2017). The secretion signal peptide of the cyanobacterial extracellular protein HesF is located at its C-terminus. FEMS Microbiol Lett.

[26] Quinlan RJ, Sweeney MD, Leggio LL, Otten H, Poulsen JCN, Johansen KS (2011). Insights into the oxidative degradation of cellulose by a copper metalloenzyme that exploits biomass components. Proc Natl Acad Sci USA.

[27] Vaaje-Kolstad G, Westereng B, Horn SJ, Liu Z, Zhai H, Sørlie M (2010). An oxidative enzyme boosting the enzymatic conversion of recalcitrant polysaccharides. Science.

[28] Harris PV, Welner D, McFarland KC, Re E, Navarro Poulsen J-C, Brown K (2010). Stimulation of lignocellulosic biomass hydrolysis by proteins of glycoside hydrolase family 61: structure and function of a large, enigmatic family. Biochemistry.

[29] Ciano L, Davies GJ, Tolman WB, Walton PH (2018). Bracing copper for the catalytic oxidation of C–H bonds. Nat Catal.

[30] Moser F, Irwin D, Chen S, Wilson DB (2008). Regulation and characterization of *Thermobifida fusca* carbohydrate-binding module proteins E7 and E8. Biotechnol Bioeng.

[31] Stanier RY, Kunisawa R, Mandel M, Cohen-Bazire G (1971). Purification and properties of unicellular blue-green algae (order Chroococcales). Bacteriol Rev.

[32] Lippi L, Bähr L, Wüstenberg A, Wilde A, Steuer R (2018). Exploring the potential of high-density cultivation of cyanobacteria for the production of cyanophycin. Algal Res.

[33] Villalobos A, Ness JE, Gustafsson C, Minshull J, Govindarajan S (2006). Gene designer: a synthetic biology tool for constructing artificial DNA segments. BMC Bioinform.

[34] Guerrero F, Carbonell V, Cossu M, Correddu D, Jones PR (2012). Ethylene synthesis and regulated expression of recombinant protein in *Synechocystis* sp. PCC 6803. PLoS ONE.

[35] Bryksin AV, Matsumura I (2010). Overlap extension PCR cloning: a simple and reliable way to create recombinant plasmids. Biotechniques.

[36] Elhai J, Vepritskiy A, Muro-Pastor AM, Flores E, Wolk CP (1997). Reduction of conjugal transfer efficiency by three restriction activities of *Anabaena* sp. strain PCC 7120. J Bacteriol.

[37] Fulda S, Mikkat S, Schröder W, Hagemann M (1999). Isolation of salt-induced periplasmic proteins from *Synechocystis* sp. strain PCC 6803. Arch Microbiol.

[38] Berepiki A, Hitchcock A, Moore CM, Bibby TS (2016). Tapping the unused potential of photosynthesis with a heterologous electron sink. ACS Synth Biol.

[39] Lundby A, Olsen JV, Gevaert K, Vandekerckhove J (2011). GeLCMS for in-depth protein characterization and advanced analysis of proteomes. Gel-free proteomics: methods and protocols.

[40] Cox J, Mann M (2008). MaxQuant enables high peptide identification rates, individualized p.p.b.-range mass accuracies and proteome-wide protein quantification. Nat Biotechnol.

[41] Zavřel T, Sinetova M, Červený J (2015). Measurement of chlorophyll a and carotenoids concentration in cyanobacteria. Bio-protocol.

[42] Kittl R, Kracher D, Burgstaller D, Haltrich D, Ludwig R (2012). Production of four *Neurospora crassa* lytic polysaccharide monooxygenases in *Pichia pastoris* monitored by a fluorimetric assay. Biotechnol Biofuels.

[43] Wood TM (1988). Preparation of crystalline, amorphous, and dyed cellulase substrates. Methods in enzymology.

[44] Westereng B, Agger JW, Horn SJ, Vaaje-Kolstad G, Aachmann FL, Stenstrøm YH (2013). Efficient separation of oxidized cello-oligosaccharides generated by cellulose degrading lytic polysaccharide monooxygenases. J Chromatogr A.

[45] Forsberg Z, Mackenzie AK, Sørlie M, Røhr ÅK, Helland R, Arvai AS (2014). Structural and functional characterization of a conserved pair of bacterial cellulose-oxidizing lytic polysaccharide monooxygenases. Proc Natl Acad Sci USA.

[46] Cannella D, Möllers KB, Frigaard N-U, Jensen PE, Bjerrum MJ, Johansen KS (2016). Light-driven oxidation of polysaccharides by photosynthetic pigments and a metalloenzyme. Nat Commun.

[47] Corbett HM, Thompson NS, Malcolm EW. Study of the carbohydrate peeling and stopping reactions under the conditions of oxygen-alkali pulping, Project 3265, Report Four: a final report to members of the Institute of Paper Chemistry. Appleton, Wisconsin: The Institute of Paper Chemistry; 1979. https://smartech.gatech.edu/handle/1853/823. Accessed 11 Mar 2019.

[48] Hildebrand A, Addison JB, Kasuga T, Fan Z (2016). Cellobionic acid inhibition of cellobiohydrolase I and cellobiose dehydrogenase. Biochem Eng J.

[49] Kruer-Zerhusen N, Alahuhta M, Lunin VV, Himmel ME, Bomble YJ, Wilson DB (2017). Structure of a *Thermobifida fusca* lytic polysaccharide monooxygenase and mutagenesis of key residues. Biotechnol Biofuels.

[50] Arfi Y, Shamshoum M, Rogachev I, Peleg Y, Bayer EA (2014). Integration of bacterial lytic polysaccharide monooxygenases into designer cellulosomes promotes enhanced cellulose degradation. Proc Natl Acad Sci USA.

[51] Guerrero F, Carbonell V, Cossu M, Correddu D, Jones PR (2012). Ethylene synthesis and regulated expression of recombinant protein in *Synechocystis* sp. PCC 6803. PLoS ONE.

[52] Matos CFRO, Robinson C, Di Cola A (2008). The Tat system proofreads FeS protein substrates and directly initiates the disposal of rejected molecules. EMBO J.

[53] Robinson C, Matos CFRO, Beck D, Ren C, Lawrence J, Vasisht N (2011). Transport and proofreading of proteins by the twin-arginine translocation (Tat) system in bacteria. Biochim Biophys Acta Biomembr.

[54] DeLisa MP, Tullman D, Georgiou G (2003). Folding quality control in the export of proteins by the bacterial twin-arginine translocation pathway. Proc Natl Acad Sci USA.

[55] Lee PA, Tullman-Ercek D, Georgiou G (2006). The bacterial twin-arginine translocation pathway. Annu Rev Microbiol.

[56] Badarau A, Firbank SJ, Waldron KJ, Yanagisawa S, Robinson NJ, Banfield MJ (2008). FutA2 is a ferric binding protein from *Synechocystis* PCC 6803. J Biol Chem.

[57] Spence E, Sarcina M, Ray N, Møller SG, Mullineaux CW, Robinson C (2003). Membrane-specific targeting of green fluorescent protein by the Tat pathway in the cyanobacterium *Synechocystis* PCC6803. Mol Microbiol.

[58] Natale P, Brüser T, Driessen AJM (2008). Sec- and Tat-mediated protein secretion across the bacterial cytoplasmic membrane—distinct translocases and mechanisms. Biochim Biophys Acta Biomembr.

[59] Lykidis A, Mavromatis K, Ivanova N, Anderson I, Land M, DiBartolo G (2007). Genome sequence and analysis of the soil cellulolytic actinomycete *Thermobifida fusca* YX. J Bacteriol.

[60] Nakai M, Sugita D, Omata T, Endo T (1993). Sec-Y protein is localized in both the cytoplasmic and thylakoid membranes in the cyanobacterium *Synechococcus* PCC7942. Biochem Biophys Res Commun.

[61] Abby SS, Cury J, Guglielmini J, Néron B, Touchon M, Rocha EPC (2016). Identification of protein secretion systems in bacterial genomes. Sci Rep.

[62] Zalucki YM, Beacham IR, Jennings MP (2009). Biased codon usage in signal peptides: a role in protein export. Trends Microbiol.

[63] Chen P-H, Liu H-L, Chen Y-J, Cheng Y-H, Lin W-L, Yeh C-H (2012). Enhancing CO_2_ bio-mitigation by genetic engineering of cyanobacteria. Energy Environ Sci.

[64] Dexter J, Dziga D, Lv J, Zhu J, Strzalka W, Maksylewicz A (2018). Heterologous expression of mlrA in a photoautotrophic host—engineering cyanobacteria to degrade microcystins. Environ Pollut.

[65] Giner-Lamia J, Pereira SB, Bovea-Marco M, Futschik ME, Tamagnini P, Oliveira P (2016). Extracellular proteins: novel key components of metal resistance in cyanobacteria?. Front Microbiol.

