# Supplementary material for: Expression and secretion of a lytic polysaccharide monooxygenase by a fast-growing cyanobacterium
Source: Biotechnol Biofuels. 2019 Apr 1;12:74. doi: 10.1186/s13068-019-1416-9 (PMC6442416; doi:10.1186/s13068-019-1416-9)
Supplement: Supplementary file 1 — Additional file 1: Figure S1. PCR analysis of S. elongatus UTEX 2973 strains TfAA10A (pDAR3) and TorA-TfAA10A (pDAR21). Figure S2. Identification of the TorA signal peptide in the pre-protein TorA-TfAA10A, found in the plasma membrane fraction, by mass spectrometry. Figure S3. Identification of the secreted TfAA10A by mass spectrometry. Figure S4. Densitometric analysis of secreted TfAA10A. Figure S5. PASC oxidation by TfAA10A. [file 13068_2019_1416_MOESM1_ESM.pdf]

Additional File 1

Expression and secretion of a lytic polysaccharide monooxygenase by a fast-growing cyanobacterium

D.A. Russo[1], J.A.Z. Zedler[1], D.N. Wittmann[1], B. Möllers[2], R.K. Singh[3], T.S. Batth[4], B. van Oort[5], J.V. Olsen[4], M. J. Bjerrum[3] and P.E. Jensen[1]

[1]Copenhagen Plant Science Centre, Department of Plant and Environmental Sciences, University of Copenhagen, Frederiksberg C, Denmark
[2]Department of Geosciences and Natural Resource Management, University of Copenhagen, Frederiksberg C, Denmark
[3]Department of Chemistry, University of Copenhagen, Copenhagen, Denmark
[4]Novo Nordisk Foundation Center for Protein Research, Faculty of Health and Medical Sciences, University of Copenhagen, Copenhagen, Denmark
[5]Biophysics of Photosynthesis, Department of Physics and Astronomy, Faculty of Sciences, Vrije Universiteit Amsterdam, Amsterdam, Netherlands

Additional File 1: Expression and secretion of a lytic polysaccharide monooxygenase by a fast-growing cyanobacterium

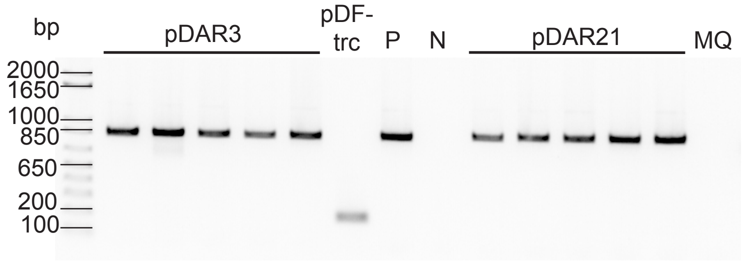

Figure S1. PCR analysis of *S. elongatus* UTEX 2973 strains *Tf*AA10A (pDAR3) and TorA-*Tf*AA10A (pDAR21). Lanes show the colonies from transformation of *S. elongatus* UTEX 2973 with plasmids pDAR3 (*Tf*AA10A), pDAR 21 (TorA-*Tf*AA10A) and an empty vector control (pDF-trc). P denotes a positive control reaction (using pDAR3 plasmid DNA), N denotes a negative control (wild-type *S. elongatus* UTEX 2973) and MQ denotes a H_2O PCR control. The following primers (5' – 3') were used for amplification of the gene of interest: trc-F (attctgaaatgagctgttga) and trc-R (atcaggctgaaaatcttctc).

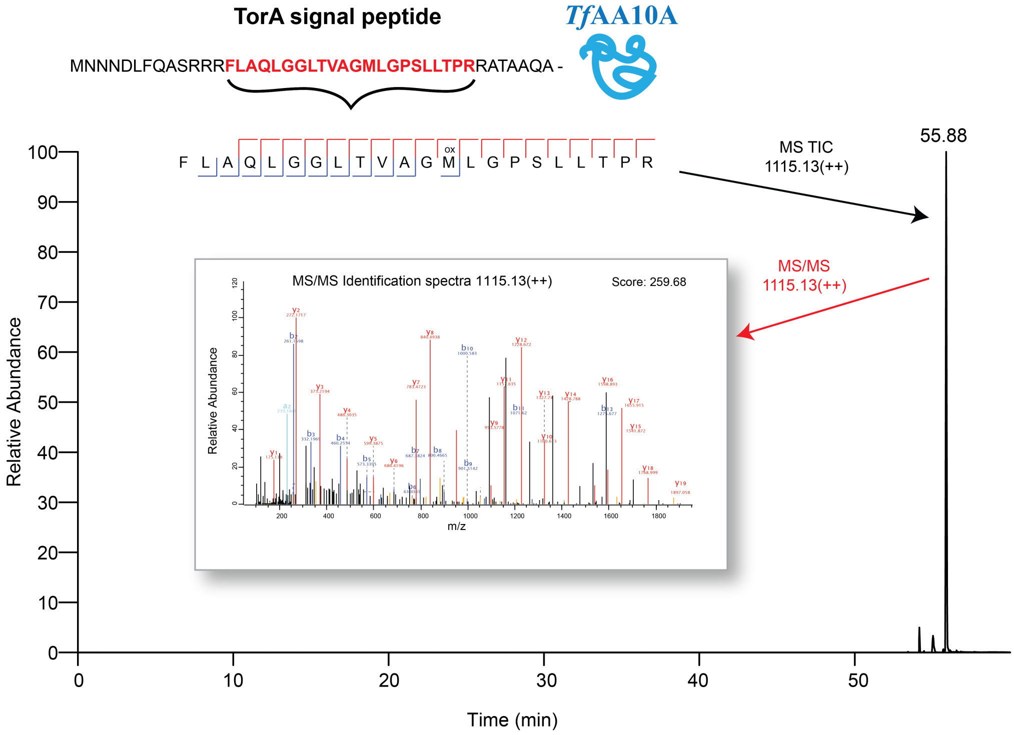

Figure S2. Identification of the TorA signal peptide in the pre-protein TorA-*Tf*AA10A, found in the plasma membrane fraction, by mass spectrometry.

Additional File 1: Expression and secretion of a lytic polysaccharide monooxygenase by a fast-growing cyanobacterium

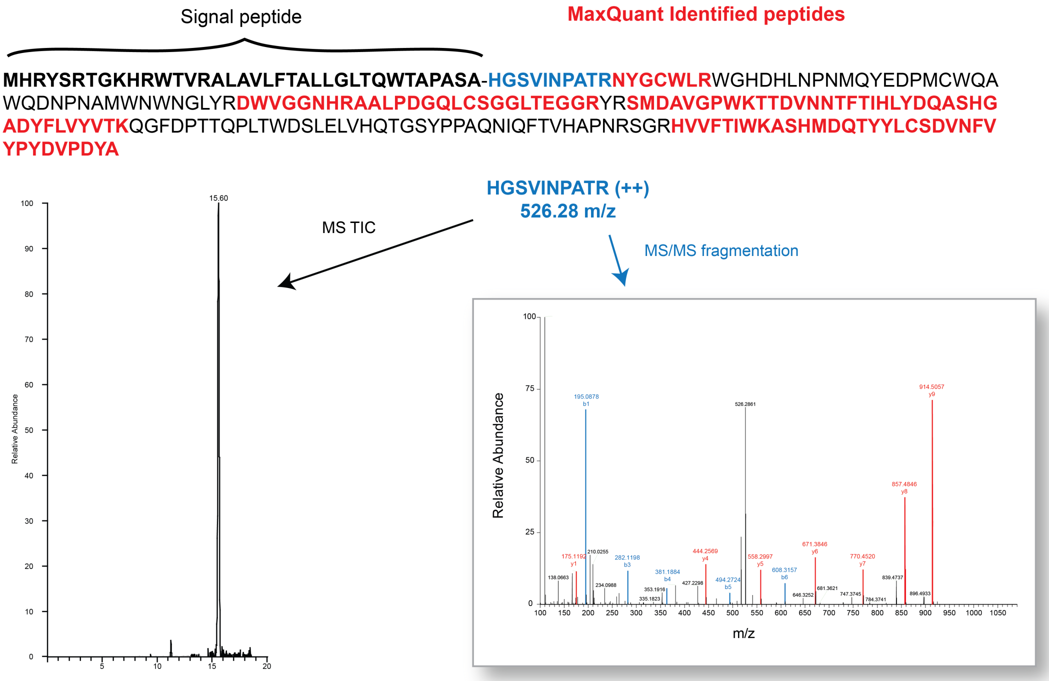

Figure S3. Identification of the secreted *Tf*AA10A by mass spectrometry. The inset highlights the identification of the mature N-terminal tryptic peptide confirming correct processing of the secreted *Tf*AA10A.

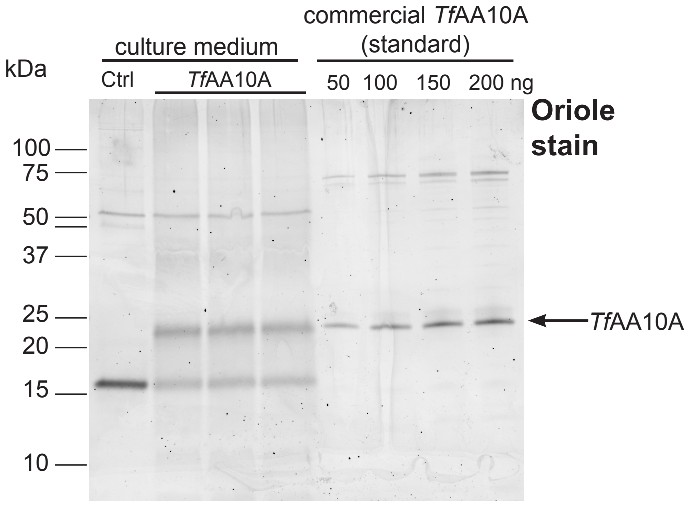

Figure S4. Densitometric analysis of secreted *Tf*AA10A. The first four lanes show culture medium loaded to an equivalent of 200 µL of cell-free medium. The subsequent four lanes show a standard curve of 50 – 200 ng of a commercial *Tf*AA10A. The *Tf*AA10A protein is indicated with an arrow. Ctrl denotes an empty vector control (pDF-trc).

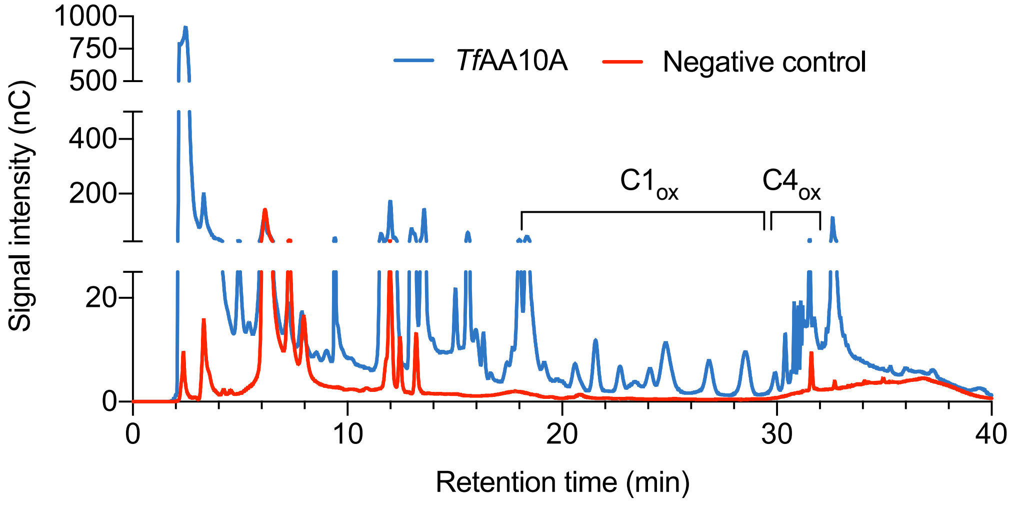

Figure S5. PASC oxidation by *Tf*AA10A. *Tf*AA10A catalyzed oxidation of PASC was performed in 50 mM phosphate buffer (pH 7.5). All reaction mixtures contained 2 mM ascorbate, 0.75% w/v PASC and 2 µg purified and reconstituted *Tf*AA10A. The negative control omitted the enzyme. Reactions were carried out at 50 °C for 24 hours. $C1_{ox}$ – C1 oxidation products. $C4_{ox}$ – C4 oxidation products. Results are representative of n = 3.
